# High Frequency of Transmitted HIV-1 Gag HLA Class I-Driven Immune Escape Variants but Minimal Immune Selection over the First Year of Clade C Infection

**DOI:** 10.1371/journal.pone.0119886

**Published:** 2015-03-17

**Authors:** Kamini Gounder, Nagavelli Padayachi, Jaclyn K. Mann, Mopo Radebe, Mammekwa Mokgoro, Mary van der Stok, Lungile Mkhize, Zenele Mncube, Manjeetha Jaggernath, Tarylee Reddy, Bruce D. Walker, Thumbi Ndung’u

**Affiliations:** 1 HIV Pathogenesis Programme, Doris Duke Medical Research Institute, Nelson R. Mandela School of Medicine, University of KwaZulu-Natal, 719 Umbilo Road, Durban, South Africa; 2 KwaZulu-Natal Research Institute for Tuberculosis and HIV, Nelson R. Mandela School of Medicine, University of KwaZulu-Natal, 719 Umbilo Road, Durban, South Africa; 3 Biostatistics Unit, Medical Research Council, 491 Peter Mokaba Road, Overport, Durban, South Africa; 4 Ragon Institute of Massachusetts General Hospital, Massachusetts Institute of Technology and Harvard University, 400 Technology Square, Cambridge, Boston, Massachusetts, United States of America; 5 Howard Hughes Medical Institute, 4000 Jones Bridge Road, Chevy Chase, MD, United States of America; 6 Max Planck Institute for Infection Biology, Charitestraße 1, Berlin, Germany; University of Cape Town, SOUTH AFRICA

## Abstract

In chronic HIV infection, CD8+ T cell responses to Gag are associated with lower viral loads, but longitudinal studies of HLA-restricted CD8+ T cell-driven selection pressure in Gag from the time of acute infection are limited. In this study we examined Gag sequence evolution over the first year of infection in 22 patients identified prior to seroconversion. A total of 310 and 337 full-length Gag sequences from the earliest available samples (median = 14 days after infection [Fiebig stage I/II]) and at one-year post infection respectively were generated. Six of 22 (27%) individuals were infected with multiple variants. There was a trend towards early intra-patient viral sequence diversity correlating with viral load set point (p = 0.07, r = 0.39). At 14 days post infection, 59.7% of Gag CTL epitopes contained non-consensus polymorphisms and over half of these (35.3%) comprised of previously described CTL escape variants. Consensus and variant CTL epitope proportions were equally distributed irrespective of the selecting host HLA allele and most epitopes remained unchanged over 12 months post infection. These data suggest that intrapatient diversity during acute infection is an indicator of disease outcome. In this setting, there is a high rate of transmitted CTL escape variants and limited immune selection in Gag during the first year of infection. These data have relevance for vaccine strategies designed to elicit effective CD8+ T cell immune responses.

## Introduction

Randomized clinical trials point to promising advances in biomedical prevention strategies against HIV-1 spread, including early treatment with antiretroviral drugs of HIV-1 infected sexual partners, the use of antiretroviral drugs in pre-exposure prophylaxis, antiretroviral microbicides and medical male circumcision [1–6]. However, the development of a safe and effective vaccine, expected to have the most significant, cost-effective and sustainable impact on HIV-1 spread remains an elusive global priority [7]. An efficacious HIV-1 vaccine will have to target the transmitted or founder virus and understanding specific genetic characteristics of successfully transmitted variants and the selection forces that shape virus evolution at the early stages of infection are critical scientific goals [8–11].

Studies have shown that HIV-1 populations at the acute phase of infection are generally homogeneous as a result of transmission of a single variant, with only a minority of infections resulting from transmission of multiple variants [12–17]. Following transmission, avoidance of host antiviral responses is a major factor influencing HIV-1 evolution [8,18–22].

HIV-1-specific CD8+ T cell responses, restricted by human leukocyte antigen (HLA) class I alleles, are thought to play an important role in the initial reduction of peak viraemia following acute HIV-1 infection [11,23–27]. In animal models of HIV-1 infection, virus-specific CD8+ T cell responses have been demonstrated to be critical for containment of virus replication and favorable clinical outcomes [28]. In addition, there is evidence that CD8+ T cell immune responses, particularly those directed against the relatively conserved HIV-1 Gag protein, are associated with reduced viral loads in chronic HIV-1 infection [29–32]. HIV-1 Gag can be an early target of cytotoxic T cell immune responses, some of which may be associated with control of viral replication [33–35]. Collectively, these data indicate that CD8+ T cell responses, particularly against relatively conserved proteins such as Gag would be desirable in protective HIV-1 vaccines. Indeed, evidence from animal models of HIV infection suggests that vaccines that induce virus-specific CD8+ T cell responses can protect against infection, reduce viral set point or attenuate disease progression [36–39].

Much uncertainty still exists over which T cell responses would contribute most to a protective vaccine [40]. Viral immune escape from CD8+ T cell immune responses is common following HIV-1 infection [41–44] which can lead to loss of viral control and disease progression [33,45–47]. Viral fitness costs associated with some immune escape variants can constrain escape or attenuate the virus, resulting in clinical benefit for the infected patient [48–53]. However, compensatory mutations that restore viral fitness are also common and eventually lead to disease progression [49,51,53,54]. Furthermore, cytotoxic T-lymphocyte (CTL) immune escape mutants transmitted to a new host can be accompanied by clinical benefit to the recipient as a result of viral attenuation and presumed loss of viral replicative capacity [20,55–59] whereas transmission of escape variants to HLA-matched recipients can be to their detriment because they are unable to mount effective CTL immune responses despite the benefit of acquiring a less fit virus [45].

A critical question relevant to HIV vaccine design and evaluation in a high prevalence setting such as KwaZulu-Natal, South Africa is the extent to which the virus is adapting to HLA class I and CD8+ immune pressure, leading to transmission and predominance of immune selected variants, and perhaps altering the impact or consequences of immune pressure as some recent studies have suggested [59–61]. In this study, we sought to better understand the multiplicity of infection, transmitted *gag* variants and immune driven viral evolution in Gag due to Gag-specific immune selection pressure in the first year of HIV-1 infection in a setting with high prevalence and incidence of HIV infection. Our data indicate that in this high incidence setting, infection is largely established by a single transmitted/founder virus and there is little evidence for immune-driven sequence variation in Gag during the first year of infection. Due to its relative conservation and the association between Gag-specific immune responses and viral control, these data encourage efforts to increase Gag selection pressure through prophylactic or therapeutic vaccines.

## Methods

### Study participants

A total of 22 antiretroviral naïve participants acutely infected with HIV-1 were enrolled in the HIV Pathogenesis Programme (HPP) Acute Infection study in Durban, KwaZulu-Natal, South Africa as described previously [62]. Briefly, at screening all subjects had detectable HIV RNA but had not yet seroconverted [negative ELISA using SD Bioline HIV1/2 Elisa 3.0 (Standard Diagnostics Inc, Kyonggi-do, Korea [3^rd^ generation]), Vironostika HIV-1 Uni-Form II Plus O v5.0 (Biomérieux, Durham, North Carolina, USA) and Western blot (GS HIV Type 1 Western blot, Biorad, Redmond, WA, USA)] according to the Centre for Disease Control and Prevention (CDC) criteria and were defined as acutely infected. The estimated date of infection was 14 days prior to screening as described previously [59,62]. Blood samples were collected at enrollment, 2 weeks, 4 weeks, 2, 3 and 6 months post infection and then every 6 months thereafter. Viral load measurements were performed at all study visits by the Roche Cobas Taqman HIV-1 Test v2.0 (Roche Diagnostics, Branchburg, NJ, USA). CD4 count enumeration was performed at all visits after enrollment using the 4-colour MultiTEST/Trucount assay (Becton Dickinson, San Jose, CA, USA) and analyzed further by flow cytometry on a FACSCalibur (BD Biosciences, San Jose, CA, USA). The median treatment-free follow-up time for the subjects was 376 days [IQR, 354–430 days], the median rate of CD4 cell decline per month calculated by linear regression was −6.79 cells/mm^3^ (IQR, −12.01 to −0.20) and the median viral load set point (mean viral load from 3 to 12 months post infection) was 4.60 log_10_ copies/ml (IQR, 3.99–4.89). Sequence-based methods were used to determine the HLA class I type [32]. Written informed consent was obtained from all study subjects and the study protocol was approved by the Biomedical Research Ethics Committee (BREC) of the University of KwaZulu-Natal.

### Gag sequencing and analysis of data

Viral RNA was isolated from 140μl of plasma samples using the QIAamp Viral RNA Extraction Mini Kit (Qiagen, Hilden, Germany). Viral RNA was then reverse transcribed using ThermoScript RT-PCR System kit (Invitrogen, Carlsbad, CA, USA) and the gene-specific primer, GagD reverse (5′-AAT TCC TCC TAT CAT TTT TGG-3′) as previously described [20]. To amplify the HIV-1 *gag* region, a nested PCR was performed with two sets of primers, GagD forward (5′-TCT CTA GCA GTG GCG CCC G-3′) and GagD reverse for the first round and GagA forward (5′-CTC TCG ACG CAG GAC TCG GCT T-3′) and GagC reverse (5′-TCT TCT AAT ACT GTA TCA TCT GC-3′) for the second round as previoulsy described [20]. The resulting PCR product was then purified using purification columns from Illustra GFX PCR DNA Gel Band Purification Kit, (GE Healthcare, Little Chalfont, Buckinghamshire, UK) and cloned into a PCR 2.1-TOPO vector (Invitrogen, Carlsbad, CA, USA). Plasmid DNA was isolated from individual randomly picked white bacterial colonies (GeneJet Plasmid Mini Prep Kit; Fermentas, Vilnius, Lithuania) and were screened for the presence of the insert with the EcoRI restriction enzyme (New England BioLabs, Ipswich, MA, USA). Sequencing was done using the ABI PRISM Big Dye Terminator Cycle Sequencing Ready Reaction kit version 3.4 (Applied Biosystems, Foster City, CA, USA).

Sequences were assembled and edited using the Sequencher Program v5.0 (Gene Codes Corporation, Ann Arbor, MI, USA). Phylogenetic relatedness to compare and evaluate intra- and inter-patient diversity was performed by Neighbor-Joining trees (with 1,000 bootstrap replicates) as implemented in Geneious v.5.0.3 created by Biomatters Ltd, Auckland, New Zealand. Branching topology was visualized in FigTree (http://tree.bio.ed.ac.uk/software/figtree). Sequence diversity was calculated using the Maximum Composite Likelihood option in Mega5.0 [63]. Subtype reference strains were obtained from the Los Alamos HIV sequence database (www.hiv.lanl.gov). Analysis of synonymous and non-synonymous substitution rates based on a set of codon-aligned nucleotide sequences was performed using the SNAP and Highlighter tool on the HIV database website [64].

### HIV-1 Gag polymorphisms

To enumerate the extent of HIV-1 CTL driven sequence variation in acute/early viral sequences we used three different approaches. We first made use of known Gag-restricted optimal HIV-1 CTL/CD8^+^ epitopes [HLA-A (n = 20), HLA-B (n = 39) and HLA-C (n = 7)] generated from studies of C-clade infected subjects [65]. Entire CTL epitopes with 5 flanking amino acids were compared to the corresponding published epitope and the percentage of variant versus consensus epitopes were enumerated for overall CTL epitopes as well as for the individual HLA-A, B and C alleles. Here, we classified ‘consensus’ as an epitope sequence that is identical to the published epitope sequence and a ‘variant’ if the epitope contained non-consensus residues. Secondly, CTL escape mutations (polymorphisms that are resistant to immune recognition which are selected by CTLs) were defined as known escape variants (as opposed to any amino acid variation) within epitopes as documented in the CTL/CD8+ epitope variant and escape mutation list at: http://www.hiv.lanl.gov/content/immunology/variants/ctl_variant.html. Thirdly, we made use of a list of HLA-associated polymorphisms, from a large number of subtype C Gag sequences from individuals in southern Africa, which were identified using methods that take into account phylogenetic relatedness of sequences, amino acid covariation and HLA linkage disequilibrium effects [66,67]. Here, HLA-associated polymorphisms were categorized as having an “adapted” (amino acids that are significantly enriched in the presence of the selecting HLA allele) or “non-adapted” form (amino acids that are significantly enriched in the absence of the selecting HLA allele) [68].

In each of the above 3 analyses, sequences were analyzed irrespective of the subjects’ HLA and then patients were categorized according to their HLA profile in order to assess whether the variants/polymorphisms were more likely to occur in the presence of HLA alleles known to select them versus those that do not, or were equally distributed irrespective of patient HLA expression profile. All three analyses were performed on viral sequences at the earliest time point and at one-year post infection.

### Nucleotide sequence accession numbers

Full-length *gag* nucleotide sequences obtained in this study have been submitted to the GenBank database under accession numbers: KM192366—KM193012.

### Statistical analysis

The relationships between multiplicity of infection, intrapatient diversity and reversion with log viral load, log viral load set point, CD4 count and rate of CD4 decline were assessed using Pearson’s correlation (for normally distributed variables) or Spearman’s rank correlation (for non-normally distributed variables). Linear regression analyses were used to generate trend lines to facilitate visualization of correlation graphs. All statistical analyses were performed using GraphPad Prism version 5.0 for Windows (GraphPad Software, San Diego, California, USA). The significance cutoff for all analyses was a *P* value of <0.05.

## Results

### Study subjects

The key demographic and clinical characteristics of the 22 subjects included in this study are presented in Table 1. The earliest available samples were at a median of 14 days post infection (interquartile range [IQR], 14–17.5 days). 17 were obtained at 14 days post infection (HIV ELISA and Western blot negative but nucleic acid positive), however, due to the unavailability of samples at screening for five participants, viral sequences were generated from these individuals at the next available time point, which ranged between 28 to 101 days post infection. The median viral load at this time point evaluated was 6.58 log_10_ HIV RNA copies/ml ([IQR] 6.00–6.95) and the median CD4 count was 423 [376–566] cells/mm^3^. The majority of the participants were female (64%) and the median age of participants at enrolment was 28 years (IQR, 24–39).

**Table 1:** Demographic and clinical characteristics of the study participants.

**PID**	**Sex**	**Age at Enrolment (yrs)**	**HLA Type**	**Estimated days since infection**	* **CD4+ T cell count/μl**	♦ **HIV-1 RNA copies/ml**	† **Western Blot**
AS1–0703	M	54	A*26:01/30:04, B*44:03/58:02, C*02:10/06:02	14	626	4, 560, 000	-
AS1–0876	F	29	A*23:01/43:01, B*15:03/44:03, C*02:10/18:00	101	309	1, 160, 000	-
AS1–0919	M	58	A*29:02/74:00, B*35:01/42:01, C*04:01/17:00	14	589	6, 280, 000	-
AS2–0016	F	28	A*26:01/30:02, B*15:18/42:01, C*17:00/18:00	14	559	31, 000, 000	-
AS2–0174	M	33	A*23:01/30:02, B*08:01/14:02, C*03:04/08:02	14	359	7, 390, 000	-
AS2–0341	M	53	A*34:02/68:01, B*15:03/58:02, C*02:10/06:02	14	492	3, 050, 000	-
AS2–0358	F	34	A*24:02/30:01, B*42:02/53:01, C*04:04/17:00	14	242	8, 560, 000	-
AS2–0483	M	27	A*30:01/34:02, B*15:03/42:01, C*02:10/17:00	14	390	33, 200, 000	-
AS2–0802	F	42	A*30:02/43:01, B*08:01/58:02, C*06:02/07:01	35	411	732, 265	-
AS2–0945	M	26	A*23:01/74:00, B*15:10/57:03, C*07:01/16:01	46	652	1, 109, 915	-
AS2–1037	F	43	A*03/34:02, B*15:03/15:10, C*02:10/03:04	34	418	12, 133, 226	-
AS21–187	F	27	A*23:01/30:04, B*15:10/82:02, C*03:02/04:01	14	448	2, 241, 051	-
AS3–0268	F	21	A*29:02/80:01, B*15:03/18:01, C*02:02/02:10	14	416	8, 640, 000	-
AS3–0369	F	28	A*66:02/68:02, B*15:10/44:03, C*03:04/07:01	14	432	3, 180, 000	-
AS3–0458	F	25	A*29:02/34:02, B*44:03/58:02, C*04:01/06:02	14	691	280, 000	-
AS3–0513	F	25	A*26:01/66:01, B*15:10/58:02, C*03:04/06:02	28	238	112, 448	-
AS3–0759	F	21	A*01:01/74:00, B*35:01/81:00, C*04:01/18:00	14	382	4, 864, 320	-
AS3–0767	F	38	A*02:02/66:01, B*18:01/58:02, C*05:01/06:02	14	424	2, 032, 173	-
AS3–0942	F	28	A*26:01/74:00, B*07:05/15:03, C*02:10/07:02	14	430	>10, 000, 000	-
AS33–182	M	22	A*29:02/30:02, B*07:02/15:03, C*02:10/07:01	14	147	563, 281	-
AS5–0710	F	16	A*01:01/34:02, B*81:00/81:00, C*08:04/18:00	14	625	61, 126	-
AS5–0968	M	22	A*23:01/30:02, B*15:10/58:02, C*03:04/06:02	14	423	>10, 000, 000	-
Median		28		14	423	3, 180, 000	

*CD4+ cell counts were not performed at screening; the values given are for 2–4 weeks post screening

♦Viral loads were performed at screening

† CDC criteria followed for the interpretation of Western Blot results

### Gag characteristics and diversity in acute or recent HIV-1 subtype C infection

To assess viral characteristics in acute or recent HIV-1 infections, full length *gag* clonal sequences from plasma viral RNA were used to determine the amino acid sequence of the viral populations at two time points. A total of 310 and 337 full-length *gag* sequences from the earliest available samples available and at approximately 1 year after infection were generated, respectively. The mean number of sequences analyzed at the earliest time point was 14 per participant (range from 12–16) and 15 sequences for the one year time point (range from 12–25).

**Fig 1:**
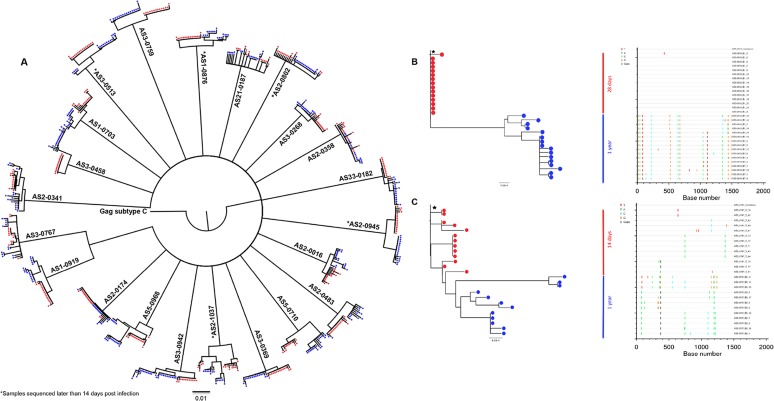
Neighbour joining trees and Highlighter plots of longitudinal HIV-1 gag diversity from recently infected individuals. (A) Neighbour-joining phylogenetic tree of longitudinal (from 14 days to 1 year post infection) *gag* sequences from 22 recently infected HIV-1 participants and consensus subtype C reference sequence from the HIV database (www.hiv.lanl.gov). Gag sequences from the earliest time point are shown in red circles and in blue circles at 1 year post infection. (*) denotes samples sequenced later than 14 days post infection (AS3–0513, AS2–1037, AS2–0802, AS2–0945 and AS1–0876 were sequenced at 28, 34, 35, 46 and 101 days post-infection respectively). (B) Participant AS3_0513 with a highly homogeneous *gag* sequence population at screening (∼28 days post infection) displaying limited structure on a tree (left) and little or no nucleotide changes from the intrapatient consensus at 28 days post infection. (C) Participant AS3_0767 infected with four closely related *gag* populations based on the clustering of sequences. Heterogeneous, multiple variant *gag* sequences population at 14 days post infection visually represented by a phylogenetic tree (left) with extensive branching topology and Highlighter plots (right) with diverse pattern of nucleotide base mutations compared to consensus. Nucleotide polymorphisms are indicated by a colored tic mark (thymine in red, adenine in green, cytosine in blue and guanine in orange) and deletions are shown by gray tics in the Highlighter plots. (★) denotes the consensus sequence obtained from the earliest time point sequences.

Sequences obtained from the 22 subjects were all classified as HIV-1 subtype C, formed independent populations with strong bootstrap support for all subjects and showed no evidence of intermingling of sequences or relatedness of virus sequences from the different participants (Fig. 1A). The majority of the *gag* sequences from each participant isolated from the earliest time point samples had monophyletic structures suggesting that they arose from a single transmitted founder virus [16 out of 22 (73%) individuals] with the remaining 6 out of 22 (27.3%) individuals showing branching topology consistent with transmission of more than one viral variant. Seventeen participants had samples available at 14 days post infection, and 12 of these displayed monophyletic viral lineages indicative of single variant transmission whereas five had heterogeneous viral populations. Interestingly, participants AS3–0513, AS2–0802, AS2–0945 and AS1–0876, sequenced at 28, 35, 46 and 101 days post-infection respectively displayed homogeneous viral populations with phylogenetic patterns indicative of single variant founder virus. Participant AS2–1037 sequenced at 34 days post infection was the exception, with a heterogeneous population consisting about 4 viral variants at the time of analysis (Fig. 1A).

Previous studies have reported that in a majority of heterosexual transmission cases a single viral variant establishes infection [8,14,15,21,69]. To further characterize the transmitted/founder virus, the Highlighter tool (Los Alamos database) was used to provide a visual representation of the viral population at each time point relative to the consensus present at the earliest time of infection, allowing discrimination between homogeneous versus heterogeneous sequences. The pattern of the Highlighter plots depicting the positions and identities of nucleotide polymorphisms; insertions and deletions across the *gag* gene were consistent with the branching topology of the phylogenetic tree constructed for each participant. Figs. 1B and 1C illustrate two examples. Fig. 1B shows the phylogeny of sequences as well as the Highlighter plot from participant AS3–0513. Of the 15 *gag* sequences obtained at estimated 28 days post infection, 14 were identical with only 1 of the 15 sequences showing a single nucleotide that differed from the consensus. However, diversification of the initial early viral populations had occurred by 1 year with the accumulation of numerous mutations when compared to the original consensus sequence. Fig. 1C illustrates a second participant, AS3–0767, who clearly exhibited a heterogeneous viral population at 14 days post infection forming more than one phylogenetic lineage. At this time point, the phylogenetic structure displayed early viral diversity with possibly 4 transmitted/founder viruses. At one year post infection, the phylogenetic structure and diversity of viral *gag* sequences obtained were more distinct and were found to form discernible clusters compared to sequences from early infection. The branching topology exhibited longer horizontal branch lengths by one-year post infection thus indicating the amount of sequence changes within *gag*.

### Impact of Gag diversity on markers of disease and evolution of intrapatient diversity

**Fig 2:**
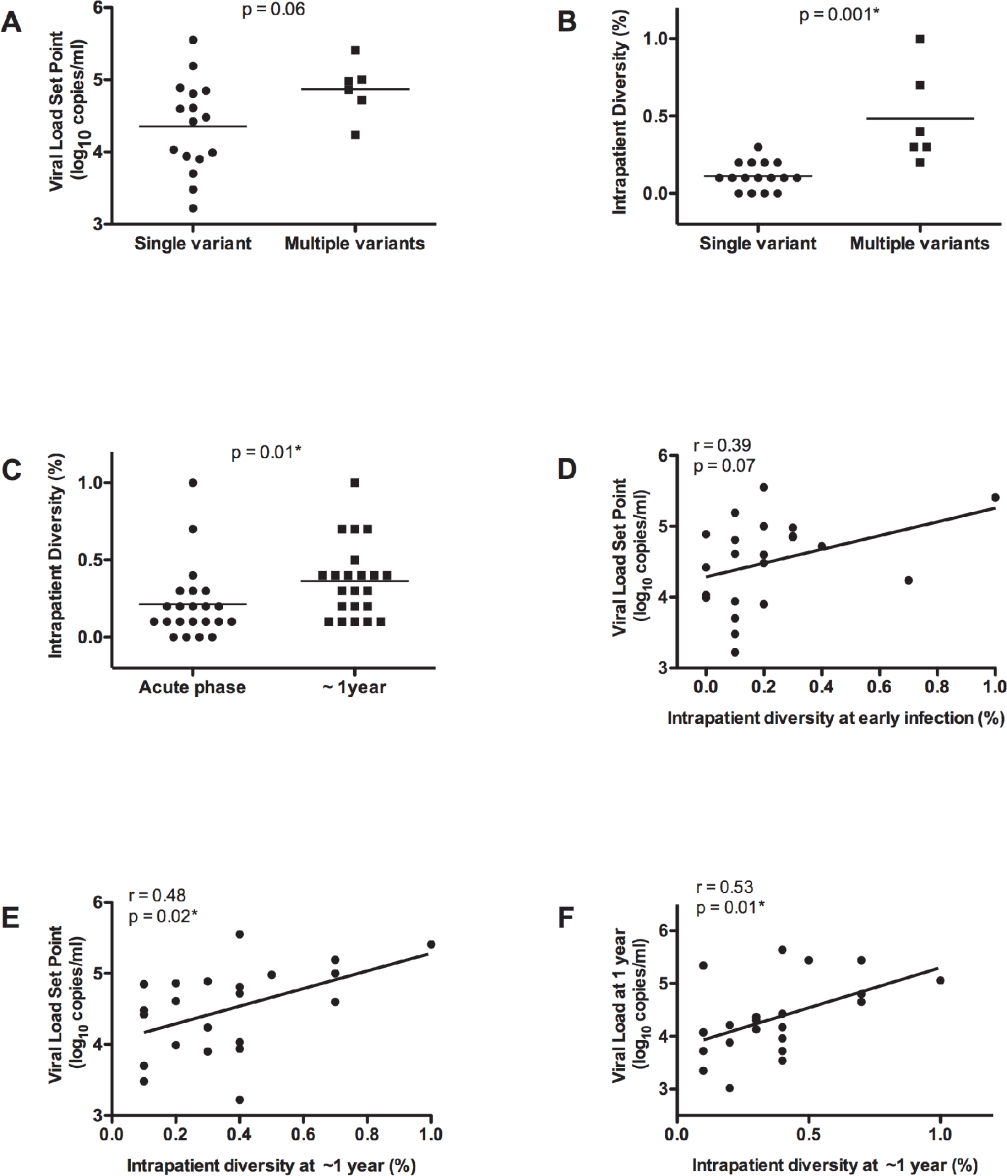
Multiple variant transmission and intrapatient diversity results in higher viral load set point. (A) Association of single versus multivariant transmission sequences versus viral load set point in individuals sequenced at the earliest time point (Student’s T test). (B) Significantly higher intrapatient diversity in individuals infected with multiple variants (Student’s T test). (C) Significantly higher intrapatient diversity within *gag* over one year of infection (Paired T test). (D) Intrapatient diversity of HIV-1 Gag at 14 days post infection correlation with viral load set point. Significant correlations of intrapatient diversity at 1 year versus viral load set point (E) and viral load at one year (F). (*) denotes statistical significant difference.

We next determined if infection with multiple founder viruses is predictive of disease progression, as defined by CD4 count and viral load, in the context of heterosexual transmission in men and women in an HIV-1 subtype C setting. When comparing single versus multiple variant transmissions there was a trend towards higher mean viral load set point for individuals infected with multiple variants (Mann Whitney test, p = 0.06) (Fig. 2A) but no difference in the rate of CD4 decline (Mann Whitney test, p = 0.13) over the first year of infection (data not shown).

In order to understand viral evolution over the first year of infection, intrapatient viral diversity was calculated for each individual. Intrapatient diversity, defined as the mean pair-wise nucleotide distance, was calculated by measuring distances between all sequences from a single individual at a single time point. As expected, intrapatient diversity was higher when comparing those with multiple versus single variant transmission/founder virus (p = 0.001) (Fig. 2B). There was a significant difference in the intrapatient diversity in early infection (median diversity = 0.15%, range 0–1%) when compared to one year later (median diversity = 0.35%, range 0.1–1%) (Student’s T test p = 0.01) (Fig. 2C). At the earliest time point there was a non-significant trend correlating viral load set point and intrapatient diversity (Spearman’s correlation r = 0.39 and p = 0.07) (Fig. 2D) although there was no association between rate of CD4 decline and intrapatient diversity (Spearman’s correlation, r = −0.16 and p = 0.48) (data not shown). Increase in intrapatient diversity was more pronounced in patients infected with single variants, from 0.1 to 0.3% (p = 0.0025) whereas participants infected with multiple variants had a non-significant increase in intrapatient diversity from 0.35 to 0.45% (p = 0.8). This finding may suggest that rapid diversification confers an advantage to the virus and therefore single variant transmissions display a significantly higher rate of diversification during the early stage of HIV infection (data not shown).

Interestingly, there was significant association between intrapatient viral diversity at one year post infection with both viral load set point (Spearman’s correlation, r = 0.48 and p = 0.02) (Fig. 2E) and viral load at one year (Spearman’s correlation, r = 0.53 and p = 0.01) (Fig. 2F) but no significant association between the CD4 count and intrapatient diversity at 1 year (Spearman’s correlation, r = −0.23 and p = 0.31) (data not shown). Overall, these data suggest that multiplicity of infection or overall viral diversity during acute infection has some impact on viral load set point but also that intrapatient diversity may be a consequence of increased viral replication capacity over the first year of infection since association between intrapatient diversity and viral load set point strengthened over time.

### Transmission or early immune-driven sequence variation during primary HIV-1 infection

HIV-1 is known to exhibit high levels of genetic variability even within individual patients, with positive natural selection by the immune system a main driver of sequence variation [64,70]. Here, our objective was to define the extent of Gag polymorphisms that exist within circulating plasma viruses during acute HIV-1 infection that could either be attributed to transmission of CD8+ T cell escape variants or early CD8+ T cell immune pressure.

**Fig 3:**
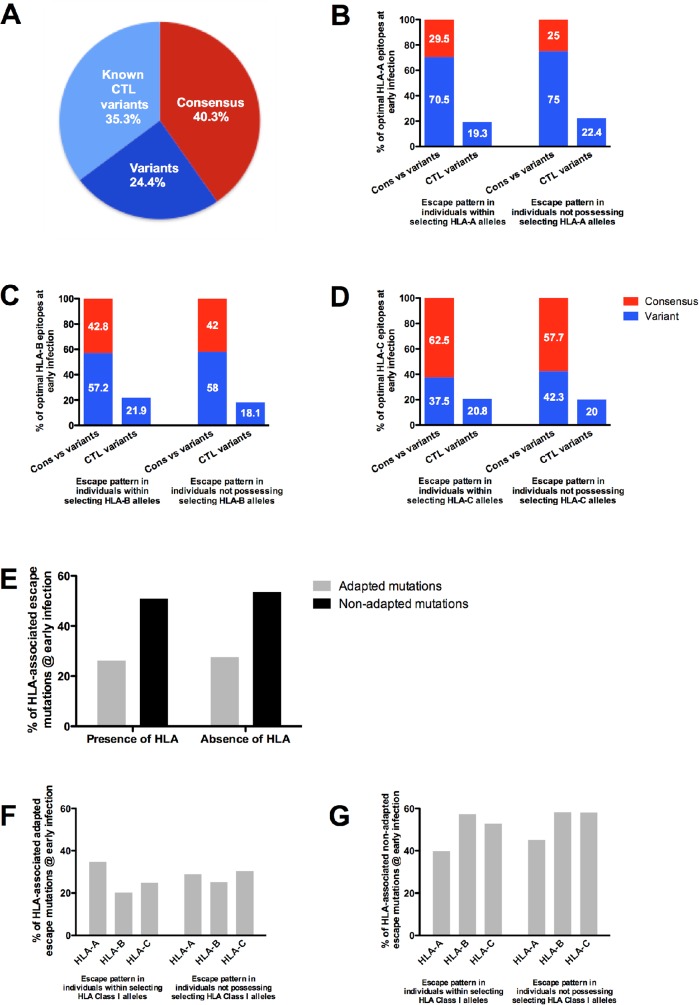
Percentage distribution of consensus and variant Gag sequence patterns in individuals at the earliest time point. (A) Percentage of consensus, variant and known CTL variants within host specific epitopes from HLA class I alleles at the earliest time of infection. Distribution of consensus, variant and percentage of variants as CTL variants within host-specific HLA restricted Gag epitopes in individuals possessing the selecting HLA-A (B), HLA-B (C) and HLA-C (D) alleles and those individuals who do not possess the selecting HLA allele at the earliest time point. (E) Overall distribution of adapted and non-adapted HLA-associated escape mutations within individuals that select and do not select for Gag polymorphisms at the earliest time of infection. Distribution of adapted (F) and non-adapted (G) mutations expressing HLA-A, HLA-B and HLA-C alleles that select and do not select for Gag polymorphisms at the earliest time of infection.

In order to do this, we first determined the percentage of consensus versus variant optimal Gag CD8+ T cell epitopes. Any defined/known HLA-restricted CTL/CD8+ epitope was classified as variant in the subject if it contained at least one amino acid variation from the published epitope sequence [65] for Clade C. Using this criteria, and when considered irrespective of host HLA alleles, the majority of known CTL epitopes (59.7%) were variant at the earliest time point (Fig. 3A). Moreover, of the earliest viruses sequenced, 35.3% contained known CTL escape variants, as defined by the LANL database.

The presence of majority variant epitopes at the earliest time point post infection may be indicative of transmission of escape variants or early immune selection pressure. If it were the latter, then variant epitopes should be enriched in persons expressing the HLA allele known to restrict that epitope. To attempt to distinguish between these two possibilities, epitopes were grouped according to the individual participant’s HLA type and analyzed according to whether the subject possessed an HLA allele that could select a polymorphism in that epitope or not. We enumerated the proportion of consensus to variant epitopes. We then quantified the fraction of CTL epitopes with amino acid substitutions known to be selected by individual HLA class I alleles (http://www.hiv.lanl.gov/content/immunology/variants/ctl_variant.html) in each subject.

The consensus and variant epitope percentages in individuals with selecting HLA-A alleles at the early time point were 29.5% and 70.5%, respectively compared to 25% and 75% in individuals not possessing the selecting HLA-A alleles (Fig. 3B). The percentage of variant epitopes that are known (defined) CTL escape variants were relatively similar between individuals possessing the restricting HLA-A allele (19.3%) and those who do not possess the restricting HLA-A allele (22.4%). Proportions of consensus to any variant epitopes were approximately even between individuals with restricting HLA-B alleles (42.8% to 57.2%, respectively) compared to those not possessing restricting alleles (42% to 58%) (Fig. 3C). The percentages of variant epitopes consisting of defined CTL variants in those individuals possessing the restricting HLA-B alleles and those who do not was 21.9% and 18.1%, respectively. The proportions of consensus to variant and known CTL variants found within patients possessing the restricting HLA-C alleles versus those not possessing the restricting HLA-C alleles were similar to the overall distributions in HLA-A and HLA-B alleles at 62.5%, 37.5% and 20.8% respectively compared to 57.7% consensus, 42.3% variant epitopes and 20% defined CTL escape variant epitopes in those not possessing targeting HLA-C alleles (Fig. 3D).

In additional analyses, we quantified escape at HLA-associated sites (HIV-1 polymorphisms that are known to be selected by specific HLA alleles) within the first year of infection with respect to the host HLA allele either towards an HLA adapted amino acid residue or away from a non-adapted amino acid residue. The total adapted codon percentage at known HLA-associated sites in the presence of the host HLA during early infection was 26.2%, compared to 27.6% in the absence of the selecting host HLA, whereas the non-adapted codons present in participants who possess the selecting HLA was 50.7% compared to 53.6% in those participants who do not possess the selecting HLA allele (Fig. 3E). Next, we analyzed each sequence for the proportion of HLA-associated polymorphisms present for each subject in the context of the patient’s HLA class I profile. At the earliest time point available, the percentages of adapted HLA-A- (35% versus 29%), HLA-B- (20% versus 25%) and HLA-C- (25% versus 30%) associated polymorphisms were comparable between those individuals possessing the selecting HLA class I allele versus those individuals who do not, with no significant differences (Mann Whitney test, p = 0.7) (Fig. 3F). Similarly, we found no significant differences within the non-adapted HLA class I associated polymorphisms between these two groups (Mann Whitney test, p = 0.4) (Fig. 3G).

Taken together, these data indicate a high rate of transmission of immune escape variants in this cohort as opposed to early immune selection for variants, since the proportion of escape variants was evenly distributed between those with targeting class I alleles versus those without these alleles.

### Immune-driven sequence variation in HIV-1 Gag during the first year of infection

The observation of a high level of transmitted variants during acute infection led us to investigate the level of immune-driven sequence variation over a period of one year. To quantify immune selection pressure within HIV-1 Gag in the first year of infection, we made use of the SNAP program to calculate the ratio of nonsynonymous (dN) to synonymous (dS) (dN/dS ratio) in sequences from the early time point compared to those from one year post infection for each participant. Interestingly, synonymous mutations were higher [median = 0.006 subs per nucleotide site (range 0–0.15)] than nonsynonymous mutations [median = 0.002 subs per nucleotide site (range 0–0.05)], median dN/dS ratio of 0.39, however not significantly different (data not shown), highlighting the conservative nature of Gag, and indicating overall the lack of selection immune pressure and strong functional constraints on Gag amino acid residue variation in this cohort.

**Fig 4:**
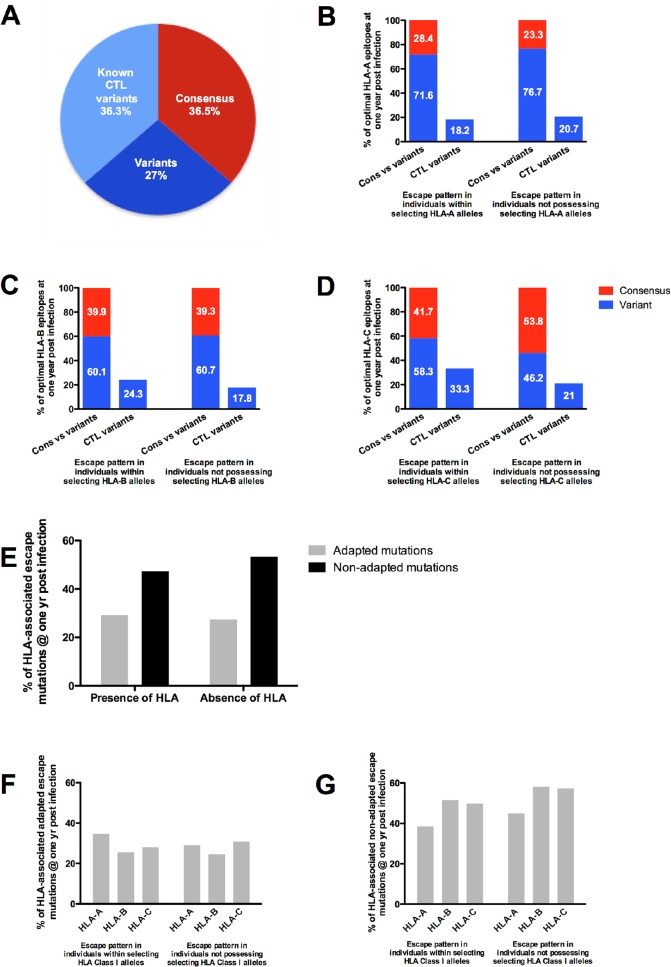
Percentage distribution of consensus and variant Gag sequence patterns in individuals over one year of HIV-1 infection. (A) Percentage of consensus, variant and known CTL variants within host specific epitopes from HLA class I alleles at one year post infection. Distribution of consensus, variant and percentage of variants as CTL variants within host-specific HLA restricted Gag epitopes in individuals possessing the selecting HLA-A (B), HLA-B (C) and HLA-C (D) allele and those individuals who do not possess the selecting HLA allele over one year of infection. (E) Overall distribution of adapted and non-adapted HLA-associated escape mutations within individuals that select and do not select for Gag polymorphisms by one year post infection. Distribution of adapted (F) and non-adapted (G) mutations expressing HLA-A, HLA-B and HLA-C alleles that select and do not select for Gag polymorphisms one year post infection.

By one year post infection, the total percentage of variant epitopes increased only slightly from 59.7% to 63.3% (Fig. 4A). In addition, the proportion of variant epitopes consisting of defined CTL escape mutations had only increased by 1% from 35.3% at earliest time point to 36.3% at one year infection. These data suggest that there was very limited CD8+ T-cell immune-driven sequence variation during the first year of infection in this cohort. In further support of this conclusion, at one year post infection there were no significant differences in the consensus to variant epitope proportions (28.4% to 71.6%, respectively) in individuals possessing the restricting HLA-A alleles, compared to the respective proportions (23.3% consensus to 76.7% variant) observed in those individuals not possessing the selecting HLA-A alleles (Fig. 4B). The proportion of epitope variants that consist of defined CTL escape variants had decreased by 1.1% in participants with targeting HLA-A alleles and by 1.7% in individuals without the selecting HLA-A alleles. Similarly, at one year the variant proportion had increased slightly from 57.2% to 60.1% and from 58% to 60.7% within the individuals possessing the restricting HLA-B alleles versus those individual who do not, with known CTL variants accounting for 24.3% and 17.8% respectively of variant epitopes, at one year post infection (Fig. 4C). Interestingly, the variant epitope proportion in individuals possessing the restricting HLA allele was found to have increased more substantially for HLA-C alleles (from 37.5% at early infection to 58.3% at one year post infection. The proportion of known (defined) HLA-C-restricted CTL epitope variants had also increased from 20.8% to 33.3% at one year post infection (Fig. 4D) in those subjects that possessed the restricting HLA-C alleles, compared to an increase from 20% to 21% in those without the restricting HLA-C alleles. These differences were not statistically significant but may be due to the small number of HLA-C epitopes (n = 7) studied compared to HLA-A and HLA-B. In additional analysis, we also calculated the percentage of individual amino acid changes that occurred within optimal Gag CD8+ T cell epitopes as well as within five amino acids flanking the epitope (as opposed to counting the percentage of consensus to variant epitopes) over one year, since mutations in these regions can impact antigen processing [71]. Interestingly, we found no difference in the individual amino acid variation that occurs within CTL epitopes and flanking regions either in the presence or absence of the selecting host HLA during the first year of infection (data not shown).

No significant differences were observed when quantifying overall total amino acid changes at HLA-associated sites for the presence of adapted and non-adapted mutations. Adapted residue increased by 3% whereas the non-adapted residues had decreased by 3.8% in those participants with the selecting HLA alleles. However, in the absence of the selecting HLA alleles the adapted and non-adapted mutations had decreased by 0.2% and 0.4%, respectively (Fig. 4E). The proportions of adapted and non-adapted HLA-A, -B and C-associated polymorphisms in each participant were comparable and we found no significant differences at the one-year time point for those possessing the selecting HLA class I allele versus those who did not (Mann Whitney test, p = 1.00 and p = 0.4 respectively) (Figs. 4F and 4G).

A comparative codon-by-codon analysis of the earliest Gag sequences (transmitted/founder virus) to matched sequences generated at one year post infection was also performed. The goal was to further interrogate whether sequence changes by one-year post infection could be attributable to immune selection pressure. Overall, the number of amino acid changes over the first year infection ranged from 0 to 16 amino acid changes within Gag. Further analysis performed to compare the relative portions of these amino acids changes occurring within and flanking targeted epitopes based on host HLA (0.72%) and those occurring in regions outside targeted epitopes (0.87%) showed no significant differences (data not shown). Collectively, these data indicate that over the first year of infection there were very limited changes in the proportions of consensus to variant epitopes and HLA-associated viral polymorphisms. Overall, there was surprisingly little evidence of CD8+ T cell immune-driven evolution of Gag sequences in this cohort during the first year of infection.

### Influence of reversion following transmission on markers of disease progression

Transmitted escape variants in a new host may revert to the consensus sequence, particularly in the absence of selective pressure in the new host due to a different HLA profile [18,20,45,72,73]. Here, reversion was conservatively defined as the presence of a CTL variant or HLA-associated polymorphism present in the early time point sequence in an individual, which is either replaced by the subtype consensus amino acid or a mutation towards a non-adapted amino acid residue in the presence of the selecting HLA allele at one year post infection.

Overall, within the first year of infection we observed limited reversion within CTL epitopes in all subjects. We identified reversions in only 3 of the 22 (13.6%) participants, with reversions occurring in 2 or 3 epitopes in these individuals (Table 2). In participant AS2–0016, reversion occurred in the HLA-B*42-HA9 and HLA-B*57-DW10 epitopes. Participant AS2–0341 had reversions occurring in epitopes HLA-B*58-SW8, HLA-B*15:10-VL10 and HLA-C*16-IM9. Interestingly, in both these participants, there was a CTL epitope reversion to consensus by one year post infection despite these individuals possessing the selecting HLA allele as noted for HLA-B*42-HA9 in participant AS2–0016 and HLA-B*58-SW8 for AS2–0341. The third participant (AS2–1037) had reversions identified at Y79F, V82I, E90A and K91R. These amino acids fall within an overlapping epitope region consisting of HLA-A*30:02-RY11, HLA-A*29-LY9 and HLA-A*02-SL9. The participant did not express any of these HLA alleles. One of the amino acid reversions (Y79F) is a defined CTL escape mutation selected by HLA*A30:02 and HLA*A02 which reverted to subtype consensus.

**Table 2:** Summary of reversion within CTL epitopes following transmission in the presence or absence of the selecting HLA type.

PID	Days PI	Optimal epitope^*a*^	Restricting HLA	HLA-association^*b*^
**AS2–0016**		EWDRLHPVHAGPIAPGQMR	B*42	+
	14	....T..............		
	463	...................		
				
		MQMLKDTINEEAAEWDRLHP	B*57	−
	14	.................T..		
	463	....................		
				
**AS2–0341**		MVHQAISPRTLNAWVKVIE	B*57/B*58	+
	14	....P..............		
	342	...................		
				
		LQGQMVHQAISPRTLNAWVK	B*1510	−
	14	........P...........		
	342	....................		
				
		SQNYPIVQNLQGQMVHQAI	C*16	−
	14	.................P.		
	342	...................		
				
**AS2–1037**		GTEELRSLYNTVATLYCVHEK	A*3002	−
	34	........F..T.......AR		
	321	.....................		
				
		EELRSLYNTVATLYCVHEK	A*29	−
	34	......F..T.......AR		
	321	...................		
				
		TEELRSLYNTVATLYCVHE	A*02	−
	34	.......F..T.......A		
	321	...................		

*a*Bold residues denotes known CTL escape mutation

*b*(+): presence of HLA-association, (−): absence of previously identified HLA association

Underlining denotes the CTL epitope in the peptide sequence

Reversions of HLA-associated polymorphisms were more common, occurring in 11 of the 22 participants (50%) (median = 1, range 1–14). Of the 28 amino acid reversions noted, 18 (64.3%) were transmitted HLA-associated polymorphisms that had reverted to consensus or a non-adapted mutation by one-year post infection in participants who did not possess the selecting HLA allele (Table 3). This high reversion rate of transmitted or founder virus HLA-associated polymorphisms in the absence of the selecting HLA suggests that these mutations were deleterious to the virus.

**Table 3:** Summary of reversion of HLA associated mutations following transmission in the presence or absence of the selecting HLA type.

PID	Gag codon position	Mutation	Restricting HLA^*a*^	HLA-association^*b*^
**AS1–0703**	91	R-K	C*06	+
**AS2–0341**	230	D-E	None	−
**AS2–0483**	242	N-T	B*57/B*58:01/C*08:02	−
**AS2–0945**	332	N-T	B*39:10	−
**AS2–1037**	79	F-Y	A*36:01/A*01/C*14/A*02:02/A*29	−
	82	I-V	None	−
	90	A-E	None	−
	91	R-K	A*74/A*66/C*06	−
	312	E-D	B*44/A*36:01/C*04	−
**AS21–0187**	228	L-M	B*49:01	−
**AS3–0268**	79	F-Y	A*36:01/A*01/C*14/A*02:02/A*29	+
**AS3–0458**	69	R-Q	None	−
**AS3–0513**	15	R-K	C*18:01	−
	228	I-M	None	−
**AS3–0942**	15	T-K	A*68:01/A*66:01/B*42:02	−
	79	F-Y	A*36:01/A*01/C*14/A*02:02/A*29	−
	106	K-E	None	−
	110	E-K	None	−
	173	S-T	A*29:02/A*26/B*81	+
	182	G-Q	B*81	−
	223	V-I	C*12/C*12:03	−
	248	T-A	None	−
	312	E-D	B*44/A*36:01	−
	315	G-N	B*53:01/B*57:03/C*17:01/B*44:03/C*04:01/B*42	−
	384	R-K	B*58:02	−
	478	S-P	B*57	−
	481	K-R	A*30:01	−
**AS5–0968**	30	Q-R	C*06	+

*a*"None" indicates no known HLA-association of mutation

*b*(+): presence of HLA-association, (−): absence of previously identified HLA association

Bold restricting HLA indicates the HLA which is positively associted with the non-adapted mutation at one-year post infection

We observed a trend toward higher viral load set point for those reverting to consensus within CTL epitopes as compared to subjects with no reversion (p = 0.08) but no difference in the rate of CD4 decline (p = 0.1). No significant differences were noted in viral load set point in individuals having reversions in the presence or absence of the selecting HLA as well as those who had not reverted (p = 0.4). However, individuals who reverted in the presence of the selecting HLA allele had a significantly higher rate of CD4 decline compared to those with no reversion (p = 0.04). Overall, these data suggest a modest impact of founder virus or transmitted virus mutations impact on markers of disease progression in this setting.

## Discussion

In this study, we characterized the dynamics of Gag early intrapatient diversity and evolution in patients within the first year of HIV-1 subtype C infection. Evolution of HIV-1 Gag transmitted/founder viruses was analyzed from as early as 14 days post infection, when adaptive immune responses are absent or minimal and at one year post infection when CD8+ T cell immune responses are already well established and expected to have an impact on virus evolution. Our data show that in subtype C infections from a high transmission setting, transmitted or founder viruses were generally homogeneous with subsequent diversification over the one year of infection. Analyses of patterns of CD8+ T cell immune pressure revealed that nearly 60% of known CTL epitopes consisted of non-consensus amino acid variants with approximately 35% containing known CTL escape variants. CTL escape variant epitopes did not differ significantly between individuals possessing the selecting HLA alleles versus those not possessing the selecting alleles, suggesting that these variants were transmitted rather than selected in the newly infected hosts. Longitudinal analysis of viral sequences revealed little sequence variation in CTL epitopes over the first year of infection, suggesting ineffective immune pressure by CD8+ T cells in this cohort during primary infection.

In this study cohort, 16 subjects (73%) had evidence of infection with a single *gag* variant. These data are consistent with a growing body of work showing that in a majority of HIV sexual transmissions only a single variant is transmitted [8,12,14,15,21,69]. Furthermore, higher intrapatient *gag* diversity at the earliest time point was associated with higher viral load set point, indicating that multiplicity of infection is associated with worse clinical outcome [74,75]. Our study highlights the need to better understand the mechanisms that underlie multiplicity of infection or higher intrapatient viral diversity during acute infection because such patients may be at a higher risk of transmitting the virus to sexual partners due to their elevated viral loads [76] in addition to the risk of higher rate of disease progression and more diverse viral quasispecies over time [77,78].

Despite the conservative nature of *gag*, we noticed evidence of viral evolution in sequences derived from plasma. Over one year the intrapatient diversity increased significantly, however further examination to measure selection argued against positive selection with a higher number of synonymous changes observed, also evidenced in elite controllers [79] indicating that evolution of HIV-1 Gag is primarily a stochastic process [80].

Another objective in this study was to better characterize and distinguish between the transmission of CD8+ T cell immune escape variants versus early immune selection. Host HLA class I-restricted CD8+ T cell responses drive HIV evolution through the selection of immune escape mutations that occur along broadly predictable pathways [56,72,81,82]. We therefore investigated whether in our cohort, published optimal CD8+ T-cell epitopes displayed evidence of immune escape patterns according to the HLA molecules expressed by the host, as an indicator of whether the immune variants were transmitted or selected in the host. Remarkably, we observed similar mutational patterns within epitopes, irrespective of the methodological approach, the patient HLA expression profile and the time point analyzed. Evaluation of HLA-driven HIV evolution employed a published list of known CTL epitopes [65] and a pre-defined HLA-associated list of polymorphisms based on a large cohort of HIV-1 subtype C infected individuals in southern Africa [66,67]. Our findings in this study showing largely unbiased representation of HLA-associated mutations in individuals with or without the selecting HLA alleles are in contrast to previous studies demonstrating that many escape polymorphisms are repeatedly selected in individuals expressing the same allele. These data strongly suggest that most CD8+ T cell immune escape variants identified during acute HIV-1 infection in this cohort were transmitted instead of selected in the newly infected host indicating that viral adaption to prevalent host HLA molecules may be occurring at a population level within this high prevalence setting [59–61]. In further support, a linked transmission pair study showed that 83.6% of polymorphisms were transmitted and that a significant fraction (17.3%) had already adapted to the linked-recipients’ HLA [83]. In addition an earlier finding from the same study also found that a surprising fraction of Gag escaped epitopes at the time of seroconversion were the same escaped epitopes present in the donor virus at the time of transmission [84].

Longitudinal analysis of Gag sequences in this study revealed diversification and viral evolution, exemplified by increase in intrapatient diversity. However, there was no substantial accumulation of CD8+ T-cell escape mutations within the first year of infection. These data are in contrast to other studies showing evidence of CTL escape as early as 25–32 days of infection [33], the first 50 days of Fiebig stages I/II [26] and 17 days following SIV infection of macaques [85]. The paucity of immune escape over the first year of infection may be due to the high transmission of immune escape variants, indicating that the virus is adapting to the prevalent HLA alleles and leading to loss of some previously protective CD8+ immune responses within the population [60,61], a plausible explanation especially for a high incidence setting such as Durban. Interestingly, our data are consistent with the recent study comparing large populations of HIV-1 infected people in Botswana and South Africa, which showed that the virus is evolving rapidly and adapting to protective HLA alleles present at the population level resulting in lower viral replication capacities [86]. Further evidence in support of the paucity of immune escape in our cohort is provided by lack of change in overall Gag-driven viral replicative fitness from the acute phase to one year post infection (data not shown).

The small sample size of our cohort calls for caution in the interpretation of the data, but the results highlight the need for additional and larger studies to address the patterns and impact of viral adaptation to prevalent host HLA class I molecules and immune escape variants fixation in the population because this may have relevance for HIV vaccine design and disease immunopathogenesis.

Despite the high transmission prevalence, only 13.6% of participants’ had reversions occurring within CTL epitopes during the first year of infection. However, in comparison 50% of individuals had HLA-associated mutations which had reverted. In HLA-B*57/B*5801-negative subjects reversion of the transmitted 242N mutation occurred between 6–24 months [20] while others reported reversions in Gag appear at a median of 62 days following seroconversion irrespective of HLA type [87]. If we take into account this time frame, it is possible that reversions from transmitted escape mutations are slow and we are noticing the very first reversions taking place. This may also represent changes in viral fitness during the early phase of infection as no or limited reversion sites detected were under immune pressure.

A number of limitations in our study should be noted. Our sample size of 22 patients limits our ability to generalize the data and further studies of recent infections in high prevalence settings are warranted to quantify the impact of immune-driven sequence variation on clinical outcomes. Our experimental approach for enumeration of transmitted/founder viruses, CTL escape mutations and HLA polymorphisms was conservative, based on standard PCR/cloning. This methodology yields similar sequence diversity results within HIV-1 populations as single genome amplification (SGA) and deep sequencing technologies [88] but may underestimate minor variants within a sample [89]. However, to ameliorate this potential bias, we performed SGA in 7/22 patients at the earliest time point post infection (data not shown). Of the seven participants that we performed SGA, one had multiple variants at transmission while the rest had single founder/transmitted variants. These results were consistent with the Sanger sequencing data.

## Conclusions

In conclusion, we provide here new information on Gag diversity and CD8+ T-cell immune-drive sequence variation in early HIV-1 subtype C infected patients in Durban, South Africa, a high prevalence and incidence setting. In a majority of individuals we observed a clear virus population bottleneck of transmission with a limited number of closely related viruses establishing a productive infection. Heterogeneous infections resulting from transmission of multiple variants were associated with higher intrapatient diversity and higher viral load set point. In addition, we found that in this setting, CTL escape variants and HLA-associated escape mutations were common among acutely infected individuals, likely selected in the transmitting partner instead of the newly infected host. Furthermore, immune escape as defined by sequence changes within Gag epitopes in the newly infected host was infrequent over the first year of infection and transmitted CTL variants did not revert easily irrespective of the HLA type of the newly infected individual. These findings may have implications for understanding the immunopathogenesis and vaccine design strategies especially for regions with severe HIV-1 epidemics.
